# Detecting Coppice Legacies from Tree Growth

**DOI:** 10.1371/journal.pone.0147205

**Published:** 2016-01-19

**Authors:** Jana Müllerová, Vít Pejcha, Jan Altman, Tomáš Plener, Petr Dörner, Jiří Doležal

**Affiliations:** 1 Institute of Botany of the Czech Academy of Sciences, Průhonice, Třeboň, Czech Republic; 2 Faculty of Science, University of South Bohemia, České Budějovice, Czech Republic; University of California Davis, UNITED STATES

## Abstract

In coppice-with-standards, once a common type of management in Central European lowland forests, selected trees (standards) were left to grow mature among the regularly harvested coppice stools to obtain construction wood. After the underwood was harvested, the forest canopy opened rapidly, giving standard trees an opportunity to benefit from reduced competition. Although this silvicultural system virtually disappeared after WWII, historical management cycles can still be traced in the tree-rings of remaining standards. Our research aims at answering the question whether tree-ring series of standard trees can be used to reconstruct past management practices. The study was carried out on 117 oak standard trees from five sites situated in formerly coppiced calcareous oak-hornbeam and acidophilous oak forests in the Bohemian Karst Protected Landscape Area, Czech Republic. The evaluation was based on the analysis of growth releases representing the response of the standards to coppicing events, and comparison to the archival records of coppice events. Our results showed that coppicing events can be successfully detected by tree-ring analysis, although there are some limitations. Altogether 241 releases were identified (49% of major releases). Large number of releases could be related to historical records, with the major ones giving better results. The overall probability of correct detection (positive predictive power) was 58%, ranging from 50 to 67%, probability for major releases was 78%, ranging from 63 to 100% for different sites. The ability of individual trees to mirror past coppice events was significantly affected by competition from neighboring trees (their number and the sum of distance-weighted basal areas). A dendro-ecological approach to the study of forest management history can serve as an input for current attempts of coppice reintroduction and for conservation purposes.

## Introduction

For centuries, coppice management was common in European lowland forests [1]. In coppice forests, shoots resprouting from stumps (coppice stools) are felled repeatedly at short intervals, providing a regular supply of firewood. Coppicing was often combined with standards (individual trees originating from seed and left to grow mature), providing building material. This form of management is referred to as coppice-with-standards [2–4]. Coppicing has a much higher disturbance frequency compared to high-forest management because of the much shorter cutting cycle. This has a profound impact on forest structure and function. After felling, insolation suddenly increases, influencing microclimate (soil temperature, moisture), evaporation, transpiration and the nutrient pool [5]. For the coppiced underwood, growth increases for a short period after cutting [6,7] (depending on the species and the site conditions), when the underwood canopy closes again [8,9]. Such cyclic changes affect the growth dynamics of standards [10,11].

The coppice cycle in Central Europe changed from 7 years in medieval times to 30–40 years in the 20th century [4,12]. Due to the use of fossil fuels and transition to modern (mostly high-forest) forestry, coppicing management virtually disappeared from most of Europe, including the Czech Republic, by the second half of the 20th century [1,7,13]. Nevertheless, its legacy can still be traced in coppice forest remnants–decaying coppice stools and standards left in overgrown coppices. Many coppiced forests as well as other cultural landscapes created by traditional management have been recognized to hold high conservation value [1,10,14].

Tree-rings have proven to be a useful tool in documenting past forest management, especially in the case of missing historical records [15]. Dendrochronology is widely used in archaeology to reconstruct past human activities and woodland management [8,16,17]. The identification of releases (i.e. abrupt increases in radial tree growth) represents one of the fundamental approaches to assess the history of forest disturbance, both natural and human-driven [18]. The sudden reduction of competition for light and nutrients in coppiced stands causes immediate growth release in standards [12,19]. In the last decades, a relatively large number of methods has been developed for release detection, which differ in pre-determined criteria and thus in the accuracy of detected releases as well as in the length of disturbance return intervals [20,21,22]. Rubino & McCarthy [22] reviewed 28 different release identification methods, and few more techniques appeared since (e.g. [23,24,25,26]).

The effects of past coppicing events on the dynamics of standard tree-ring increment remain little explored. We found only a few studies, which demonstrated varying levels of success. While [11] considered coppicing a severe disturbance event, [10] showed only a mild effect of felling, which increased the growth of the remaining standard trees by 20% for about 7 to 9 years. [11] achieved high correspondence of detected releases with historical coppicing events, whilst [27] found post-coppicing growth releases at only one of the four forest sites studied.

The objective of our research was to evaluate the possibilities of dendroecological methods to reconstruct coppicing history, assessing (a) the ability of tree-rings to reflect historical coppice events by comparing detected releases to historical evidence of coppicing, and (b) the relative importance of competition in predicting the magnitude of release as a response of standard trees to coppicing events. Our study was performed in abandoned coppice-with-standard oak-hornbeam and acidophilous oak forests in the Bohemian Karst Protected Landscape Area (PLA). These forests were regularly coppiced until WWII, and they still retain their original structure. This enabled us to distinguish standards from overgrown coppice shoots. Standard trees were old enough to cover several coppice cycles, and a number of historical forestry documents are available at least partly in the archives to reconstruct coppicing events.

## Materials and Methods

A field permit was obtained from the Nature Conservation Agency of the Czech Republic.

### Site description

**Fig 1:**
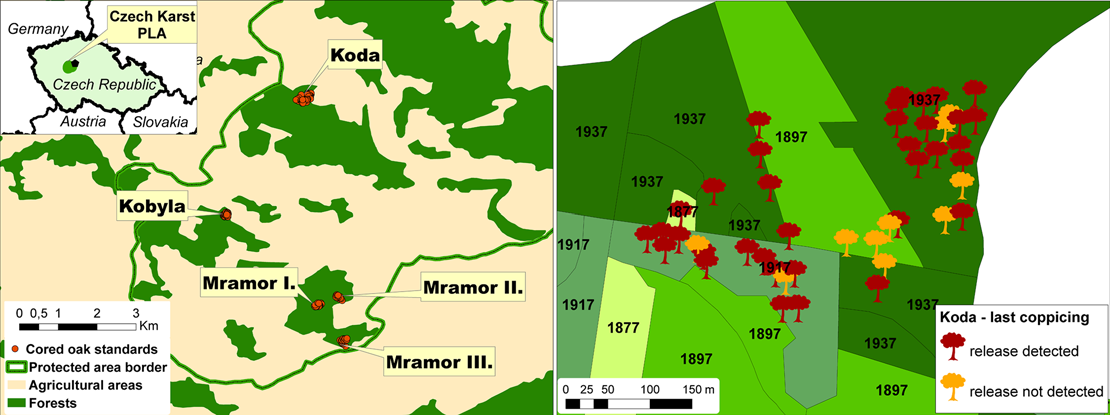
Study area (left) and a detailed view of the Koda stand with a year of the last coppice harvest marked (right part).

The study area is located in the Bohemian Karst PLA, Czech Republic (49.9134106N, 14.1093536E; Fig 1), and is protected since 1972. It is both a geologically and biologically valuable landscape southwest of Prague, with characteristic karst formations. The bedrock consists mainly of limestone. Mean annual temperature in the area is ca. 8.5°C, mean annual precipitation 530 mm, altitudes vary between 208 and 499 m a.s.l. The area has been inhabited by humans for millennia. It consists of a mosaic of arable fields, villages, and forests (38% of the PLA). The forests tend to be located on hilltops, and are often isolated. The bedrock, specific geomorphology, warm and dry climate with a xerothermophilous flora, and the role of humans have resulted in high biodiversity including protected flora and fauna, often light demanding species dependent on the traditional management.

Since human population has always been relatively large and the need for firewood high, the vast majority of the forests in the Bohemian Karst have been intensively managed. Forests composed of hornbeam, lime and oak were suitable for coppicing or coppicing with standards. Both types of management were practiced until WWII and since then the formerly coppiced trees have grown up to the height of the standards and thus completely closed the canopy.

For this study, we selected the following forest complexes (hills): Koda National Nature Reserve, Kobyla Nature Reserve and Mramor. Koda as well as parts of Kobyla and Mramor are under Natura 2000 protection. Except for a part of Mramor, the hills consist of limestone bedrock and are covered by Calcaric Cambisols and oak-hornbeam forests. The southern part of the Mramor complex consists of sandstone covered by Dystric Cambisol, and is overgrown by less dense oak forests with an admixture of *Pinus sylvestris* (3%). Within these forest complexes, we selected five mature forest stands with well-preserved oak standards on both limestone and sandstone bedrock dominated by *Carpinus betulus* and *Quercus petraea* agg. These were Koda, Kobyla, and Mramor I, II and III (Fig 1; S1 Table). Although all trees were about the same height, standards were clearly distinguishable from overgrown coppice stools by their typical appearance of wide crowns and branches starting at about 1/3 of the stem.

### Data collection and analysis

#### Sample preparation and measurements

Cores were collected from 117 oak standards at breast height (1.3 m, one core each) using increment borers (Haglof, Sweden) in 2012–2014. We measured their diameter at breast height (DBH), and recorded their exact geographical position using a differential GPS Trimble PathFinder Pro XRS. Cores were placed in straw and transported to the dendrochronological laboratory of the Institute of Botany in Třeboň. A thin layer of wood was sliced off from each core using a core microtome [28] to highlight the tree-rings and allow for the inspection of reaction wood and other abnormal properties. Ring widths were measured to the nearest 0.01 mm using the TimeTable measuring device interfaced with PAST4 software [29] and an Olympus SZ51 stereomicroscope. Individual tree-ring series were visually cross-dated using the pattern of wide and narrow rings [30] and verified using the PAST4 [29].

#### Detection of past coppice events

Coppicing affects tree growth similarly to natural forest disturbances (e.g. [23,31]), which means that its history can be traced by the identification of releases. To reconstruct coppice events, we tested two methods of release detection from the tree-rings: (i) the radial-growth averaging (GA) criteria [32], and (ii) boundary-line (BL) criteria [33]. In GA method, the average radial growth over the preceding 10-year period, *M*_*1*_ (including the target year), and the average radial growth over the subsequent 10-year period, *M*_*2*_ (excluding the target year) are calculated. The percentage growth change (*%GC*) is obtained by: *%GC* = ((*M*_*2*_*–M*_*1*_)/*M*_*1*_*)* * 100. In BL method, mean radial growth in prior 10-years is calculated, and the BL is constructed by dividing prior growth data into 0.5 mm segments with the top ten values of %GC averaged within each segment. Finally, the curve is fitted to all positive segment averages using function with the highest R^2^ [23]. The base dataset and formula for the BL followed [11]. To include information about the severity of the disturbance, i.e. coppicing, detected releases were divided into categories of moderate and major to reflect mild and severe disturbance, respectively. The minimum thresholds for both moderate and major releases were adopted from [32,33]. Calculations were performed with R software, version 3.1.3 [34] using the TRADER package [20].

#### Comparison to forest archives

Forest archives were investigated for records of coppicing. Forest maps were digitized and georeferenced. Information on coppice events (calculated from the forest age in a given year) was derived from the map legend and corresponding forest management plans using ArcGIS 10.1 software [35].

In the past, forest stands in the study area were divided into relatively small forest compartments (2 to 4 ha on average depending on the map source). Usually more than one compartment was coppiced in any given year, Fig 1). From historical records it follows that all underwood in a certain forest compartment was harvested at once or more precisely the compartments were delineated to always include the coppice underwood of the same age. It means that the compartment borders could change following the harvest to reflect the actual forest age. The pattern of coppice events therefore differed in space and time even within the same analyzed stand.

Historical records of coppicing and detected release events were compared, with two types of error (c.f. [26,36]): (i) commission error, in which a release was identified with no coincident coppice event in the archives, i.e. false detection, and (ii) omission error, in which coppicing was recorded in the archives but no release was detected. To assess the accuracy of tree-ring analysis for coppice event detection, we measured for each cored tree the sensitivity (measure of commission error, ratio of correctly detected and all archival coppice records) and positive predictive power (PPP; measure of omission error; ratio of correctly detected and total detected releases) [36,37]. A five-year difference was allowed to compensate for both variation of individual growth patterns and possible errors in the archives. Documentation in the archives was not complete. Some records were missing, and the accuracy of releases detected in such time periods could not be verified. Such releases were excluded from the detection accuracy assessment.

#### Effects of competition

Since all studied forest stands were left to grow without management measures for the last 60 years, and both coppice underwood and seedlings could develop spontaneously, we assume that present stand density reflects the site natural conditions and can therefore explain part of historical tree growth variability, such as in [11]. To analyze the effects of competition by neighboring trees, all trees of DBH over 4 cm at a distance of up to 10 m from the cored standard trees were identified and measured (their distance and DBH), the same way as in [11] and [38]. Statistical differences between the study sites were tested using analysis of variance. To analyze the effect of competition, several indices of crowding intensity (CI) were calculated to account for both density- and size-related neighbor effects and for intraspecific vs. interspecific interference. Because the effects of neighbors decrease with distance from a subject tree, the neighbor tree basal area was weighted by its distance to the cored tree [39]. CIs based on density were calculated as (i) number of all stems within 10 m from the cored standard, (ii) separately for dominating oak, hornbeam, and others, and (iii) separately for multi- or single-stem neighbors. CIs accounting for size-related neighbor effect were (iv) the sum of basal areas of all neighbors weighted by their distance (DWBA) from the cored tree, (v) DWBA divided by the species, and (vi) DWBA divided by the stem structure. Competition and tree growth parameters were related to the response of standards to coppice events (number of releases, mean growth change—MGC, sensitivity and PPP values) using generalized linear models in R [34]. Predictor variables were selected by step-wise selection of model receiving the best Akaike’s Information Criterion [40,41] using MASS package in R [42].

## Results

### Tree-ring analysis

**Fig 2:**
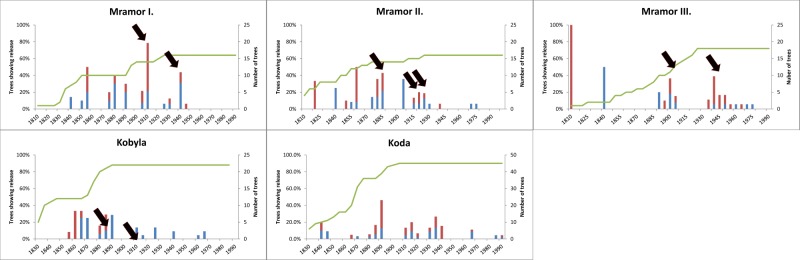
Moderate (blue) or major releases (red columns) detected using the GA method displayed in 5-year intervals. Years of coppicing recorded in archives are marked by black arrows. The green line indicates sample depth.

Altogether 117 oak standards from five forest stands were used for the reconstruction of coppice cycles. The average age of oak standards was 150 years, ranging from 90 to 215. The BL method showed unbalanced results with overestimation of releases at Koda site (S1 Fig, S2 Table) and was therefore excluded from the further analysis. By the GA method, altogether 241 releases were identified, 49% (123) of which were determined as major (Table 1, Fig 2, S2 Table). Standard trees experienced 2.1 release events on average. The highest value was found at Mramor II. (2.6 releases per tree), the lowest at Mramor III. (1.8 releases per tree). Regarding the release intensity, trees at Kobyla site experienced markedly less major releases (0.5) compared to other sites (1.1–1.2 major releases per tree, Table 1). The frequency of the detected releases indicates that coppicing was performed in 20- to 40-year intervals (with the exception of Kobyla, where major releases were detected only in 1894/5). Moreover, release reconstruction revealed that majority of severe events occurred before WWII (97%).

**Table 1:** Summary of cored trees and release events (GA method).

	Mramor I.	Mramor II.	Mramor III.	Kobyla	Koda	Total
No. of cored oaks	16	16	18	22	45	117
Age of cored oaks	90–196	97–210	97–215	116–176	106–196	90–215
DBH of cored oaks	129–208	144–215	120–202	125–225	111–205	111–225
No. of releases detected	40	42	33	42	84	241
Average No. of releases per tree	2.5	2.6	1.8	1.9	1.9	2.1
Average No. of major releases per tree	1.2	1.2	1.1	0.5	1.1	1.0
Proportion of trees experiencing some release	87.5%	100.0%	88.9%	90.9%	97.8%	94.0%
Proportion of major releases	47.5%	45.2%	60.6%	28.6%	57.1%	49.0%

### Concordance with historical records

Silvicultural system applied in all five studied stands during the analyzed time span (last 230 years) was coppicing with standards. Forests were coppiced in 25–30 year cycle. In the 1890s, the cycle was extended to 40 years and after WWII. coppices were left to overgrow. Exact counts of standards are only available for Koda stand in 1864, and partly in 1944. Such sparse records do not allow assessment of changes in standard density.

For the historical analysis, ten forest maps and five forest management plans and surveys were found in the archives (S3 Table). Concordance of some detected releases could not be verified due to the missing archival records (no records between 1892–1944 for Koda and prior 1902 for Mramor and Kobyla forests, S3 Table). This accounts for 34% of releases (45 major and 38 moderate), that were excluded from the analysis.

The degree of compliance between detected release and historical coppice events varied (Table 2). While some coppice events remained undetected, some generated high number of releases with up to 86% compliance (expressed by sensitivity). 58% of detected releases agreed with the archives (i.e. PPP), ranging from 58% at Kobyla to 67% at Koda site (Table 3). 47% of archival coppice events were detected by tree-ring analysis (i.e. sensitivity), ranging from 35% at Kobyla to 58% at Koda site (Table 3). The highest proportion of major releases (around 60%) was found at Mramor III. and Koda sites (Table 1). Regarding release type, major releases showed comparably higher agreement to archival records compared to moderate ones (78% of major releases agreed, ranging from 63 to 100% for different sites).

**Table 2:** Agreement between historical forest archives and releases detected by GA method expressed by sensitivity. Percentages of trees showing release ± 5 years from the year of coppicing are shown.

Site	Coppice event	Trees experiencing coppicing	Trees detecting coppicing (major/moder.)	Sensitivity (± 5 years)
Mramor I.	1911–15	14	9/3	85.7%
	1935–40	15	0/1	6.7%
Mramor II.	1888–93	13	5/6	84.6%
	1917–25	13	2/3	38.5%
	1935	8	0/1	12.5%
Mramor III.	1900–01	13	2/2	30.8%
	1940	14	8/1	64.3%
Kobyla	1894–95	21	5/6	52.4%
	1915	22	0/4	18.2%
Koda	1845	7	1/2	42.9%
	1861–63	7	0	0.0%
	1916	14	7/4	78.6%
	1934–44	31	14/6	64.5%

**Table 3:** Accuracy of coppice detection from tree release (GA method) compared to historical coppice records (± 5 years). Positive predictive power (PPP) express probability of correct detection and is the inverse of the commission error accounting for false detections, whereas sensitivity is the inverse to the omission error accounting for undetected coppice events.

Site	Coppicing detected (major/moder.)	False detection (major/moder.)	Coppicing not detected	PPP	PPP (major release)	Sensitivity
Mramor I.	9/4	4/6	17	57%	75%	43%
Mramor II.	8/8	3/9	14	57%	73%	53%
Mramor III.	10/3	6/7	16	50%	63%	45%
Kobyla	5/10	0/11	28	58%	100%	35%
Koda	26/10	5/13	26	67%	82%	58%

### Effects of site conditions

**Fig 3:**
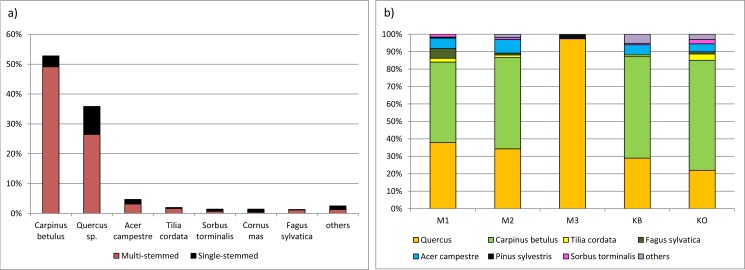
Species composition within a 10 m radius of the cored standards, divided into single- and multi-stemmed (a), and by study site (b). Sites were dominated by hornbeam and oak except the sandstone-based Mramor III site, where hornbeam was missing and pine formed 3% of the neighbours.

**Fig 4:**
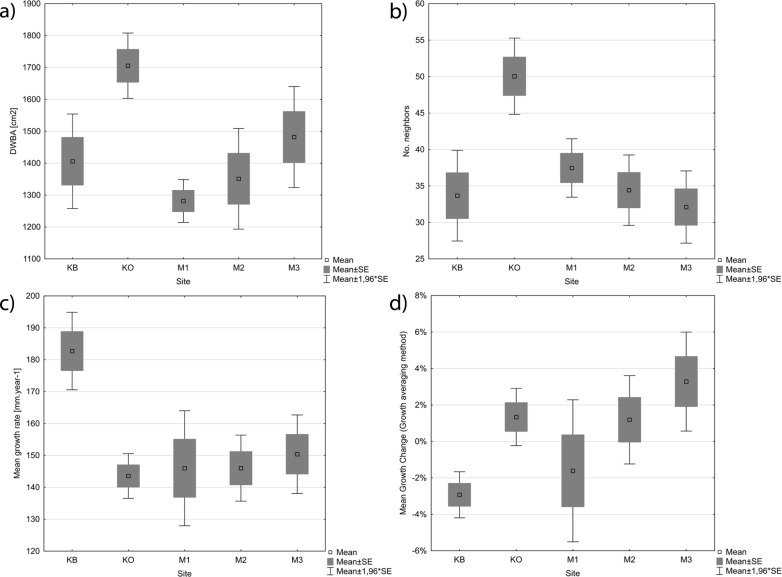
Competition from neighbouring trees within a 10 m distance from cored standard trees expressed by distance-weighted basal area (a), and number of neighbouring stems (b), mean growth rate (c), and mean growth change identified by GA method. KO = Koda, KB = Kobyla, M1–3 = Mramor I–III. Significance tests are summarized in S4 Table.

At a distance of up to 10 m from the cored oak standards, we recorded 42.6 stems with sum basal area of 9623 cm^2^ per standard on average. 84% of the stems were coppice shoots, i.e. multi-stem structured (Fig 3A, Fig 4, S2 Table). Stems of coppice origin were mostly hornbeam (*Carpinus betulus;* 59%) and oak (*Quercus petraea* agg.; 31.5%). Individual seed-originated trees were mostly oaks (59%), with about one quarter of young stems with DBH < 50 cm. Single stems of hornbeam made up 23%, and were mostly young (three quarters). Some species, such as *Pinus sylvestris* and *Betula pendula* were represented only by trees of seed origin. Koda showed significantly higher neighbor density (expressed by both DWBA and number of stems; Fig 4; S4 Table) compared to other stands.

Except for the silicate-based Mramor III stand, the most abundant species were hornbeam (46–63%) and oak (22–38%). Other species forming 3–8% (in order of abundance) were *Acer campestre*, *Tilia cordata*, *Fagus sylvatica*, *Sorbus torminalis*, *Acer platanoides*, *Pinus sylvestris*, and *Corylus avellana*. The sandstone-based Mramor III stand was very monotonous in species composition, covered by oak (97%) with an admixture of pine (3%; Fig 3B).

Tree response to the coppicing expressed by the number of releases detected in tree rings as well as the MGC were significantly influenced by the intensity of neighboring competition—number of neighbours showed positive and their DWBA negative correlation (Table 4). Number of releases was also positively influenced by cored tree age, whereas MGC was negatively influenced by mean growth rate. Intraspecific competition from hornbeam played more important role compared to the interspecific one for both response variables. The structure of stands (single-stemmed neighbors) played significant role in MGC giving the same pattern as previous, i.e. positive correlation with the number of single-stemmed trees and negative with their DWBA. For number of releases the role of the stem structure was not significant. Ability of standards to reflect coppice events (expressed by sensitivity and PPP values) was positively related to the MGC, whereas the relationship with the neighbor competition was not clear (Table 4).

**Table 4:** Models testing effect of growth parameters and neighboring competition on the tree response to the release (number of releases and mean growth change), and the accuracy of coppice detection from tree-ring analysis (expressed by sensitivity and PPP). Significance was tested in generalized linear models using software R version 3.1.3. Best predictor variables were selected by stepwise model selection by Akaike’s Information Criterion using MASS package in R.

Variables of initial models (No. releases and MGC): MGR; No. neighbors (all, oaks, hornbeam, other); DWBA (all, oaks, hornbeam, other); age					
**Number of releases**	Estimate	Std. Error	t value	Pr(>|t|)	** **
(Intercept)	-0.2987461	0.5185749	-0.576	0.5657	** **
age	0.016471	0.0034024	4.841	4.29E-06	***
DWBA hornbeam	-0.0014463	0.0006678	-2.166	0.0325	*
No. hornbeam	0.0226908	0.0116399	1.949	0.0538	**.**
				AIC: 328.19	** **
**Model I. for MGC**	Estimate	Std. Error	t value	Pr(>|t|)	** **
(Intercept)	7.46E-02	4.22E-02	1.768	0.0799	**.**
MGR	-3.81E-04	1.88E-04	-2.031	0.0447	*
DWBA hornbeam	-6.19E-05	2.47E-05	-2.505	0.0137	*
No. all neighbors	7.89E-04	4.40E-04	1.793	0.0757	**.**
DWBA oak	-2.03E-05	1.45E-05	-1.405	0.163	
				AIC: -326.59	
Variables of initial model (MGC): MGR; No. neighbors (all, single-stemmed, multi-stemmed); DWBA (all, single-stemmed, multi-stemmed); age					
**Model II. for MGC**	Estimate	Std. Error	t value	Pr(>|t|)	
(Intercept)	4.76E-02	2.84E-02	1.676	0.09662	**.**
DWBA single-stemmed	-5.33E-05	1.86E-05	-2.874	0.00487	**
No. single-stemmed	3.03E-03	1.12E-03	2.706	0.00791	**
MGR	-2.57E-04	1.75E-04	-1.465	0.14587	** **
				AIC: -331.18	
Variables of initial models (sensitivity and PPP): MGC; No. releases; MGR; No. neighbors (all, oaks, hornbeam, other); DWBA (all, oaks, hornbeam, other); age					
**Sensitivity**	Estimate	Std. Error	t value	Pr(>|t|)	
(Intercept)	0.914319	0.208031	4.395	2.60E-05	***
MGC	2.512293	0.632153	3.974	0.000128	***
age	-0.002966	0.001243	-2.385	0.018825	*
No. oaks	-0.006481	0.003534	-1.834	0.069373	**.**
No. all neighbors	0.003364	0.002191	1.535	0.127586	** **
				AIC: 104.73	** **
**PPP**	Estimate	Std. Error	t value	Pr(>|t|)	** **
(Intercept)	0.548567	0.0638904	8.586	7.02E-14	***
No. hornbeam	0.0085018	0.0048387	1.757	0.0817	**.**
MGC	1.2113676	0.7090305	1.708	0.0904	**.**
DWBA hornbeam	-0.0004111	0.0002778	-1.48	0.1418	** **
				AIC: 132.47	** **

Signif. codes: 0 ‘***’0.001‘**’0.01‘*’0.05‘.’0.1‘ ‘1.

Abbreviations: DWBA—distance-weighted basal area of neighboring trees; MGC—mean growth change; MGR—mean growth rate; PPP—Positive Predictive Power.

## Discussion

Our study covered a relatively diverse area of both calcareous and acidophilous oak forests in Bohemian Karst, Czech Republic, enabling us to reconstruct the 100–200-year history of formerly coppiced oak forests growing in different environmental conditions. This time span included several coppice events, but was too short to capture shifts in coppice cycles, which usually happened in the 17^th^ and first half of the 18^th^ century [4,12]. Coppice cycles reflected in tree-rings agreed with the general trend of coppice abandonment by WWII or even earlier [13]– 40% of the cored trees experienced the last coppicing in the 1920s.

We are aware of the fact that sample size was imbalanced. Ideally a larger sample size for all sites (such as in Koda) would have been better, but the number of preserved standard trees in the area was limited (all available standards were cored).

### Choice of release detection method

The success of coppice reconstruction from tree-rings was sensitive to the method selected for the release detection. From the two methods tested, the BL criteria [33] was found unsuitable for our study for several reasons, mainly because of bias in release number among the sites. For this method, we applied BL previously calculated in [11]. Although large datasets of ring width measurements (app. 50,000) are supposed to be necessary [21], a lot of studies successfully fitted BL with a considerably lower number of tree-ring width measurements (see Appendix in [20]), because BL tends to be universal for individual species across stands. However, in some cases construction of local BL is necessary; e.g. for *Picea abies* [43]. Such heterogeneity in release response can be caused by differences in genotype, site conditions, range position, disturbance history [21,23] and biogeoclimate [44]. Prior using the known BL, its fit to the analyzed data should therefore be checked [43,45,46]. In our case it seems that application of BL from study of [11] was not suitable, as visible specifically in Koda, where it probably overestimated releases due to substantial environmental differences between the analyzed site and the reference site used to develop the BL. Moreover, GA method shows higher sensitivity for release detection compared to BL. One of the most important steps before applying the GA technique is selection of appropriate criteria and parameters. In this study, we decided to follow the criteria as suggested by [32] instead of more conservative ones (e.g. [47]), not sensitive enough to detect releases of old-age, overstory trees [32,47]. Used criteria afford the detection of local or subtle canopy disturbances [32], which can play important role in the study forest ecosystem and increase the similarity between reconstructed disturbance chronology and historical records.

### Concordance to archival records

Tree-ring analysis showed app. 50% agreement with historical coppice records. The major releases contributed most to the tree-ring/archive concordance. Accuracy of detection varied substantially among coppice events from mostly detected to almost/completely undetected, and was positively related to the MGC, meaning that with stronger response of the tree to the coppicing higher accuracy to archival records was achieved. Accuracy of detection was also indirectly negatively affected by mean growth rate via its correlation with MGC. In this study, we achieved lower concordance than 90% as referred to in [11], however, study [27] was far less successful in coppicing detection, proving post-coppicing growth releases at only one of the four study sites. The lower accuracy compared to [11] could have been caused by the fact that the analyzed area was much larger and more diverse in both natural conditions and site history. Including both calcareous and acidophilous oak forests not only enabled us to broaden the environmental variability of formerly coppiced oak forests, but also resulted in more complexity, as the site conditions (competition) were proven to significantly affect the ability of trees to mirror coppice history.

Possible causes of errors were: (i) false detection (omission error) because a historical record on coppicing was missing, or the release was caused by small scale (illegal) cutting or environmental variation only; (ii) coppicing not detected (commission error) because historical records were not precise, or the magnitude of the release was too small to be detected. We did not find any records on extreme climatic events such as wind storms or ice/snow damage nevertheless such events may not necessarily be recorded if small scaled.

In error assessment, we have to consider the imperfection/incompleteness of archival records and inaccuracies in forest maps. In case of a time gap in archival records, releases were not included into the analysis to avoid bias, however such releases did not influence accuracy. As mentioned in [11], historical records on coppicing were connected to calendar years while felling was carried out in the winter season or sometimes in two consecutive years. Although some coppice events documented in the archives were detected with very high accuracy, some were not detected by tree-ring analysis at all. This fact might signify inconsistency and inaccuracy in archival records. For example, unrecorded small coppice events or illegal cutting, manifested as moderate releases, would reduce the PPP value. The age of forests was recorded both in forest maps and in forest management plans. However, the given dates did not always agree. In this case the oldest record of a given event was considered. To overcome these inaccuracies, the ± 5 year interval was applied. As stated in [4], some archival records on tree harvesting were related to plans that were not followed too rigidly. Our study sites comprised several forest compartments with different timings of coppicing. Various archival materials were used to acquire dates of past coppice events. Differences in their quality could also have contributed to differences in detection accuracy.

Historical forest records usually concentrate on underwood coppice, and do not provide details on possible neighboring standard tree felling. Because of the size of standard trees, felling would have had great influence on the whole forest ecosystem. However, due to their density (distance to each other), the direct influence on neighboring standing trees would not have been so pronounced. Moreover, standard and underwood felling usually happened at the same time, only magnifying the disturbance effect.

### Important role of competition

Part of historical variability in the tree growth could be explained by current neighbor density on the stand, such as in [11]. In our case, the magnitude of release (represented by MGC and number of releases) was significantly influenced by the neighbor competition. Higher was the number of smaller trees (especially hornbeam) in the standard vicinity, stronger was its response to the disturbance event, such as coppicing, suggesting that competition from larger trees (more single-stem trees of the seed origin) reduce the reaction of tree to the coppicing event. We did not find any clear pattern in relation of coppice detection accuracy and the neighbor competition.

### Implications for nature conservation

Comparison to documented coppice events in forest archives allowed us to assess the suitability of dendro-ecological methods for a reconstruction of coppicing history. Our results proved that coppicing events can be detected by tree-ring analysis through the identification of growth releases with relatively high accuracy. Considering the mentioned limits, our reconstruction can be regarded robust enough to be applied for investigation of management history. Currently, growing efforts to re-establish coppicing for ecological purposes generate needs for detailed knowledge on the history of coppicing. Attempts to reintroduce coppice management for conservation purposes should take into account patterns of past coppicing and its abandonment [4]. Thanks to the coppicing legacies preserved in the tree-rings, dendroecological approach can provide such detail insights into the tree history and can serve as an important input for coppice reintroduction for conservation purposes.

## Conclusions

Our research provides a detailed dendroecological assessment of historical coppice events reflected in the tree-rings of standard trees. Tree-ring data proved to be a suitable source of information about past forest management (coppicing). Nonetheless, we are aware of the limitations of the method, e.g. neighbor competition seems to influence the ability of tree-rings to mirror past coppice events. Our study contributes to the understanding of forest dynamics and the knowledge of coppice management history, and may serve as an important input for current attempts of nature conservation to re-establish coppice forest management.

## Supporting Information

S1 Fig Moderate (blue) or major releases (red columns) detected using the BL method displayed in 5-year intervals. Years of coppicing recorded in archives are marked by black arrows. The green line indicates sample depth.
(TIF)

S1 Table Site characteristics (XLSX)

S2 Table Data used in the analysis. (XLSX)

S3 Table Summary of archival sources. (FM—forest map, FMP—forest management plan, FS—forest survey SOA—State Regional Archive, PLA—Protected Landscape Area)
(XLSX)

S4 Table Differences in competition of neighboring trees up to 10 m from the cored standards (ANOVA followed by Tukey HSD test). DWBA = distance-weighted basal area. Values in bold are significant at p < 0.05.
(XLSX)
