# Supplementary figures and images for: Detecting Coppice Legacies from Tree Growth
Source: PLoS One. 2016 Jan 19;11(1):e0147205. doi: 10.1371/journal.pone.0147205 (PMC4718612; doi:10.1371/journal.pone.0147205)

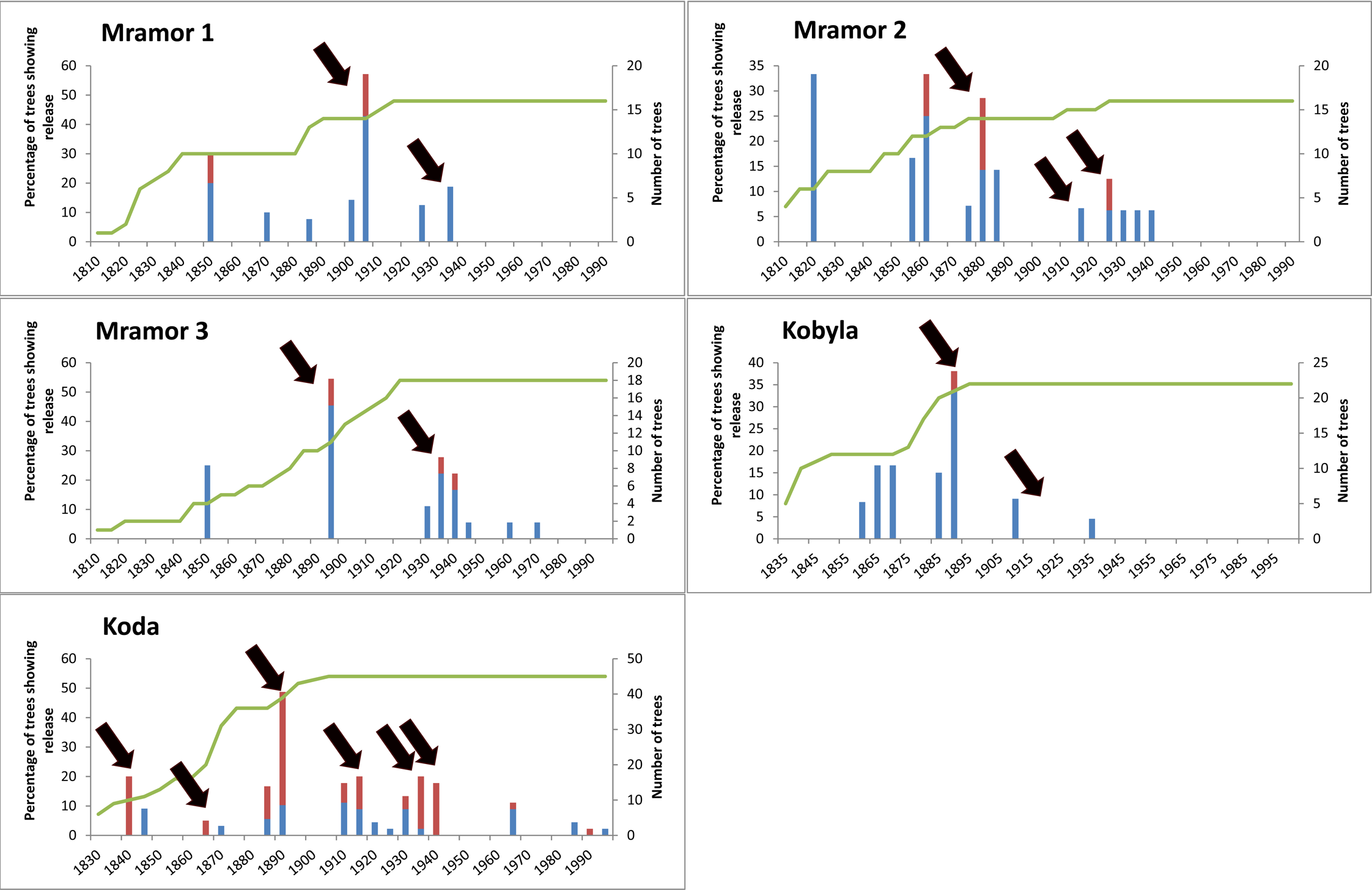

Supplement: S1 Fig — Years of coppicing recorded in archives are marked by black arrows. The green line indicates sample depth. (TIF) [file pone.0147205.s001.tif]
